# Eccentric Exercises on the Board with 17-Degree Decline Are Equally Effective as Eccentric Exercises on the Standard 25-Degree Decline Board in the Treatment of Patellar Tendinopathy

**DOI:** 10.3390/medicina59111916

**Published:** 2023-10-29

**Authors:** Vladimir Knež, Damir Hudetz

**Affiliations:** 1 Special Hospital for Medical Rehabilitation Varaždinske Toplice, 42223 Varaždinske Toplice, Croatia; 2 Department for Orthopaedic Surgery, University Hospital, “Sveti Duh”, Sveti Duh 64, 10000 Zagreb, Croatia; ortohud@gmail.com; 3 Department for Traumatology and Orthopaedics, University Hospital Dubrava, 10000 Zagreb, Croatia

**Keywords:** patellar tendinopathy, eccentric quadriceps exercise, decline board, rehabilitation

## Abstract

*Background and Objectives*: Patellar tendinopathy is one of the most significant problems in jumping and running athletes. Eccentric quadriceps exercise has been introduced into the therapy of patients with patellar tendinopathy in order to avoid weakening the tendon during rehabilitation. The use of decline boards with a decline angle of 25° has been the cornerstone of therapy over the last two decades. Biomechanical studies have suggested that an equal or potentially better outcome could be achieved with lower angles of decline (up to 16°). *Materials and Methods*: In this present research, we compared the effects of two various decline board angles on the clinical outcome of patients treated for patellar tendinopathy by performing eccentric quadriceps exercises. Patients were randomly allocated into two groups: patients practicing on the standard board with a 25° decline, and patients practicing on the 17° decline (*n* = 35 per group). *Results*: After 6 weeks of exercise, we found a significant improvement in all the clinical scores (VISA-P score, KOOS score, Lysholm Knee Questionnaire/Tegner Activity Scale, and VAS scale) of treated patients. However, there was no significant difference between the patients who performed eccentric quadriceps exercises on the standard 25° decline board and those exercising on the 17° decline board. A smaller additional degree of improvement was visible at the end of the follow-up period (at 12 weeks), but, again, no statistical difference could be detected between the investigated groups. We conclude that both treatment options provide similar short-term and midterm benefits regarding improvements in pain and clinical scores. The improvement in clinical scores does not depend on age, sex, BMI, or the professional sport of the patient. *Conclusions*: Our findings encourage changes in the decline angle of the board in the case of a patient’s discomfort in order to achieve better compliance without affecting the recovery.

## 1. Background and Objectives

Patellar tendinopathy is one of the most significant problems in jumping and running athletes. It is a common overuse injury characterized by inflammation and pain in the affected knee, which can interfere with athletic participation and lead to a significant decrease in performance [1,2,3]. Although the pathophysiology of its occurrence is still not completely elucidated, the principal cause of its occurrence is thought to be repetitive small stress injuries obtained during training or game performance [4,5]. Its prevalence depends on the sport and age of the athlete, as well as the amount of time spent training. Various studies estimate that it may affect 5–25% of athletes [6,7,8].

Initially, the treatment consisted of rest, activity modification, and the administration of anti-inflammatory drugs. However, during the last few decades, it has become generally accepted that the treatment of this condition should not only be focused on the removal of inflammation and pain but also on tendon healing and strengthening, with the return of the athletes to their adequate professional activities as the final aim of this treatment [9,10,11]. In order to achieve this aim, eccentric quadriceps exercise, which is performed to avoid the weakening of the tendon, has become the standard treatment option for the rehabilitation of patients with patellar tendinopathy. However, various ways to perform squats during these exercises have been described, and no squat has been generally accepted as superior to others [12,13]. In an interventional study performed on a relatively small number of patients, Purdam et al. reported that patients who performed squats on a 25° decline board recovered better than patients practicing on the flat position. Patients practicing on the decline board experienced a greater decrease in VAS scores, and a greater number returned to sports activities [14]. Subsequent biomechanical studies provided evidence that performing squats on the decline board indeed modifies the pattern of muscle activity in comparison to flat board exercises. For example, Lee et al. demonstrated that, when performing decline board squats, greater activity of rectus femoris with lower activities of tibialis anterior and gastrocnemius are achieved than in flat board squats [15]. Richards et al. also reported greater activity of rectus femoris and lower activity of tibialis anterior but found greater activities of gastrocnemius and biceps femoris, suggesting that this stabilizes the knee against an anterior displacement of the femur on the tibia that occurs during the decline board squat [16].

The most common decline board used In eccentric quadriceps exercise is a standard 25° decline board [17,18,19]. However, there is a gap in the knowledge about whether the change in the decline angle would affect the usefulness of the practice. In a biomechanical study, Zwerver et al. investigated the influence of various decline board angles (0°–30°) on the patellofemoral contact force and concluded that any angle greater than 15° ensured a significant increase in the maximum patellar tendon force. These findings suggest that any decline board with an angle between 15° and 30° can be used, whichever feels most comfortable to the patient [20]. In addition, the aforementioned biomechanical study by Richards et al. showed the maximum activity of knee extensors when using a 16° decline board, suggesting that the maximum benefit of the exercise can be attained at angles as low as 16° and that no additional mechanical advantage is gained by increasing the angle of declination to 24°. However, this conclusion is based solely on biochemical performance and has not been confirmed in studies focused on the clinical outcome assessing the pain and functionality of patient knees. Long-term clinical outcome-oriented studies were, to the best of our knowledge, conducted only for standard 25° decline boards and demonstrated its superiority compared to flat surfaces [21]. Although the minimal angle of decline necessary for the beneficial effect suggested by biomechanical studies appears to be 15 or 16°, we decided to select 17° to avoid a potentially marginal angle. Therefore, in this present randomized study, we compared the clinical outcomes between the two groups of patients performing exercises on a 17° and 25° decline board. Our main findings show an equally good recovery in both groups of patients, confirmed by the improvement of various standardized clinical scores, suggesting that a decrease in the decline angle to 17° provides equally good treatment benefits as a standard 25° decline board.

## 2. Materials and Methods

### 2.1. Patients

Seventy patients diagnosed with patellar tendinopathy were recruited into this study at the Department of Orthopedics, University Hospital “Sveti Duh”, Zagreb, Croatia. Patients younger than 18 years, patients with history of previous injury or treatment (ligament, meniscus, fracture, etc.), and patients who declined to sign informed consent were excluded from this study. This study was conducted in accordance with the Declaration of Helsinki and approved by the Ethics Committee of University Hospital “Sveti Duh” (protocol code 012557, and by the University of Zagreb Faculty of Kinesiology protocol code: 6/2017, date of approval: 23 March 2017). After enrollment of the patients, written consent was obtained from all of the participants, and the patients were randomly divided into the following two groups: patients practicing on the standard board with 25° decline angle and patients practicing on the 17° decline angle. Each group consisted of 35 patients who were randomly allocated to the groups via the sealed envelope system [22]. At the beginning of this study, 70 sealed envelopes were prepared (35 for each treatment) and mixed. After patients consented to participate in this study, one of the envelopes was selected and opened to allocate the patients into the 17° or 25° treatment group. At the beginning of this study, all patients filled out the Victorian Institute of Sport Assessment Patella (VISA-P) questionnaire and the Knee Injury and Osteoarthritis Outcome Score (KOOS) questionnaire. Functional knee activity was assessed using the Lysholm Knee Questionnaire/Tegner Activity Scale, and patients assessed their pain using the Visual Analog Scale (VAS). The details of the scores used are explained below. The same set of analyses was performed at the end of a 6-week therapy period and after an additional 6 weeks. Therefore, the total follow up of the recruited patients was 12 weeks after the beginning of the treatment.

### 2.2. Victorian Institute of Sport Assessment Patella

The VISA-p questionnaire was used to assess the severity of the symptoms and functional ability in patients. The questionnaire consists of 8 items (four on pain associated with functional activity, two on ability to perform functional activity, and two on ability to perform sport) subdivided into three sub-questions. The total score can range from 0 (the worst) to 100 (no pain at all) [23,24].

### 2.3. Knee Injury and Osteoarthritis Outcome Score

The KOOS questionnaire consists of 42 questions subdivided into five outcome items (pain, symptoms, activities of daily living, sport and recreation function, and knee-related quality of life). Scores are then transformed to a 0–100 scale, with zero representing extreme knee problems and 100 representing no knee problems at all [25,26].

### 2.4. Lysholm Knee Questionnaire/Tegner Activity Scale

The Lysholm Knee Questionnaire/Tegner Activity Scale is a functional, activity-oriented scale that consists of eight questions on limping, using cane or crutches, locking sensation in knee, giving way sensation in knee, pain, swelling, abilities to climb stairs and squat. As in previously described scales, a score of 100 indicates no problem at all, and theoretical worst possible score is zero [27,28].

### 2.5. Assessment of Pain by Visual Analog Scale

Patients assessed their pain on a standard 10 cm line; a score of 100 indicates highest possible pain, and a score of zero indicates no pain at all [29].

### 2.6. Statistical Analysis

For numerical variables, data are presented as mean ± standard deviation (SD). The differences between the groups were tested using Student’s *t* test for independent samples, or paired Student’s *t* test, as appropriate. Chi-square test was used to compare categorical data. All tests were two-sided, and *p* < 0.05 was considered statistically significant. Statistical analysis was performed in GraphPad Prism version 6 for Windows (GraphPad Software Inc., La Jolla, CA, USA).

## 3. Results

Demographic data for both groups of patients are shown in Table 1. There was no statistical difference in age or sex between the groups. Furthermore, the groups did not differ in BMI, body weight, or sport participations. The initial VAS score, VISAP-1 score, Lysholm/Tegner, and KOOS score indicated that a similar degree of pain and dysfunctionality of the knee was present in both groups of patients (no significant difference, *p* > 0.05).

For continuous variables, data are shown as mean ± standard deviation, and *p* values were calculated using an independent *T*-test.

For categorical data, the number of patients with percentage is shown, and *p*-values were calculated using Chi-square test.

### 3.1. Both Treatment Options Significantly Improved the Function of the Knee without Differences between the Treatments

**Figure 1:**
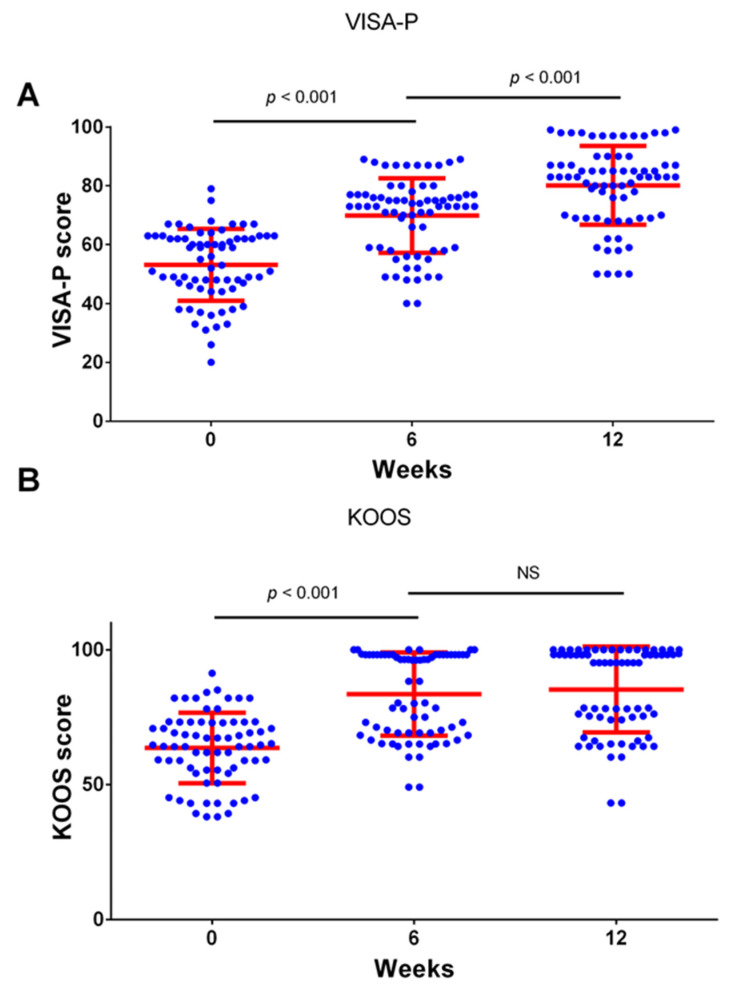
Clinical scores for VISA-P (**A**) and KOOS (**B**) at the beginning of this study, after 6 weeks of treatment, and after 12 weeks. Clinical scores were determined at indicated time points. Dots represent individual values, and lines represent mean with standard deviation. Comparisons were made using paired *t*-test with Bonferroni correction for multiple testing (*n* = 70 patients). NS—not significant. *n* = 70 patients.

**Figure 2:**
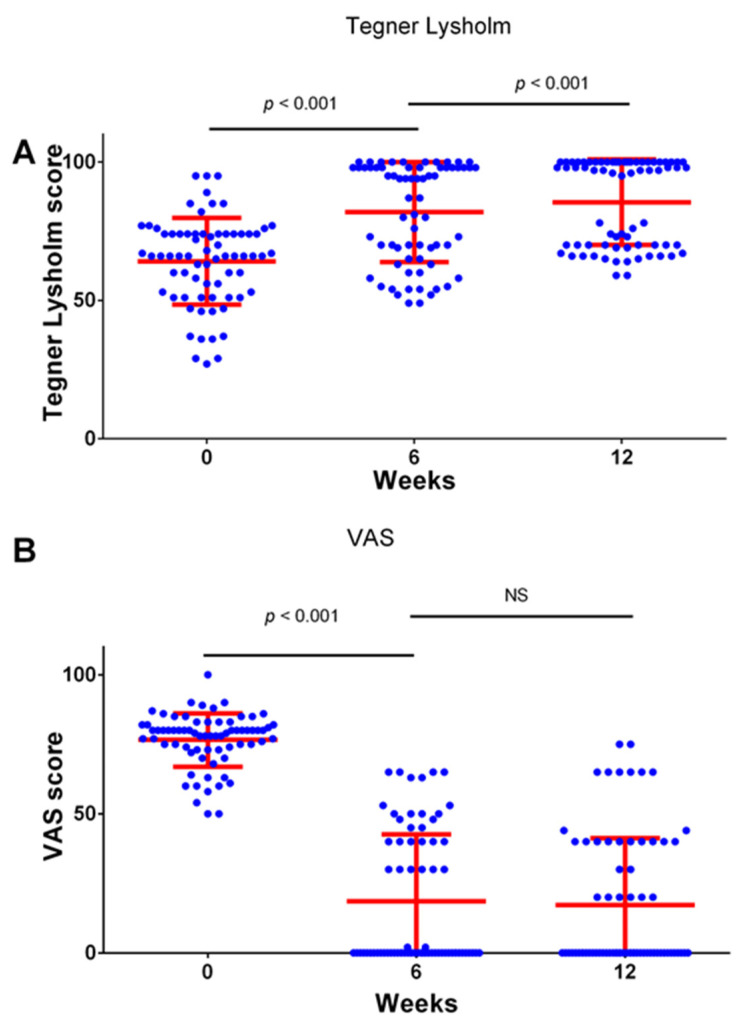
Clinical scores for Lysholm Knee Questionnaire/Tegner Activity Scale (**A**) and VAS score (**B**) at the beginning of this study, after 6 weeks of treatment, and after 12 weeks. Clinical scores were determined at indicated time points. Dots represent individual values, and lines represent mean with standard deviation. Comparisons were made using paired *t*-test with Bonferroni correction for multiple testing (*n* = 70 patients). NS—not significant. *n* = 70 patients.

The analysis of all 70 participants as a single group during the investigated three time points showed that the Visa-P score improved significantly during the treatment period from 53.15 ± 12.23 to 69.89 ± 12.66 after six weeks (*p* < 0.001, paired *T*-test) with further improvement to 80.11 ± 13.39 (*p* < 0.001) occurring after the additional 6 weeks (Figure 1A). The KOOS score improved from 63.57 ± 13.09 to 83.55 ± 15.45 during the first six-week period (*p* < 0.001, paired *T*-test), and no significant further improvement was recorded after the additional six weeks at 85.27 ± 15.92 (*p* > 0.05, Figure 1B). Similarly, the majority of the improvement in the Lysholm/Tegner Activity Scale occurred during the first six weeks (64.13 ± 15.68 vs. 81.93 ± 18.09, *p* < 0.001,) with a smaller further improvement after the additional six weeks (81.93 ± 18.09 vs. 85.54 ± 15.46, *p* < 0.001, Figure 2A). The degree of the pain measured using the VAS score decreased significantly during the first six weeks (76.57 ± 9.58 vs. 18.6 ± 24.04, *p* < 0.001, Figure 2B), and there was no significant further improvement after the additional 6 weeks (*p* > 0.05. Figure 2B).

**Figure 3:**
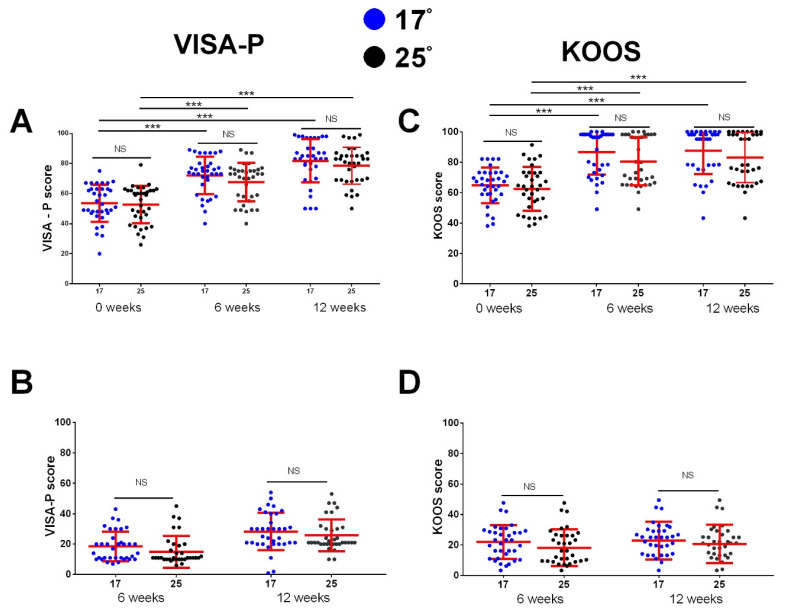
Improvement in clinical scores for VISA-P and KOOS at the beginning of this study, after 6 weeks of treatment, and after 12 weeks. Clinical scores were determined at indicated time points. Improvements in clinical scores (**B**,**D**) were determined by subtracting values measured at 6- and 12-week time points from 0 weeks. (**A**,**C**) shows comparison between the 17° and 25° group for VISA-P and KOOS scores at different time points (comparison performed using independent *T* test, NS for all comparisons); for each group, the values between the different time points were tested using paired *T*-test with Bonferroni correction (*p* < 0.001 for all comparisons, indicated by ***). In (**B**,**D**), the difference in improvement in clinical scores between the groups is shown (independent *T* test with Bonferroni correction). Dots represent individual values, and lines represent mean with standard deviation (*n* = 35 patients per group). *** *p* < 0.001, NS—not significant. *n* = 35 patients per group.

**Figure 4:**
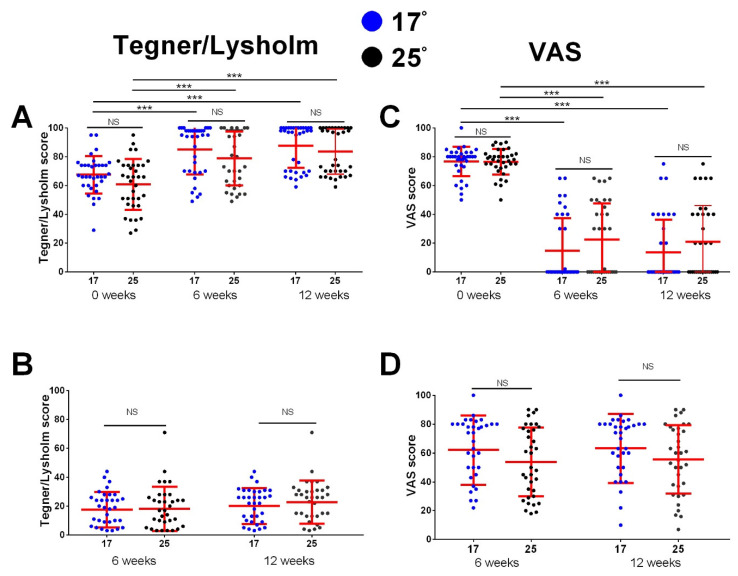
Improvement in clinical scores for Lysholm Knee Questionnaire/Tegner Activity Scale and VAS score at the beginning of this study, after 6 weeks of treatment, and after 12 weeks. Clinical scores were determined at indicated time points. Improvements in clinical scores (**B**,**D**) were determined by subtracting values measured at 6- and 12-week time points from 0 weeks. Calculated values for VAS score improvement are negative but, for clarity, were multiplied by −1. (**A**,**C**) shows comparison between the 17° and 25° group for Lysholm Knee Questionnaire/Tegner Activity Scale and VAS score at different time points (comparison performed using independent *T* test, NS for all comparison); for each group, the values between the different time points were tested using paired *T*-test with Bonferroni correction (*p* < 0.001 for all comparisons, indicated by ***). In (**B**,**D**), the difference in improvement in clinical scores between the groups is shown (independent *T* test with Bonferroni correction). Dots represent individual values, and lines represent mean with standard deviation (*n* = 35 patients per group). *** *p* < 0.001, NS—not significant. *n* = 35 patients per group.

The comparison of improvements revealed no significant difference between the two treatment options. In both groups of patients, significant improvement in VISA-P, KOOS, Lysholm/Tegner Activity Scale, and VAS score occurred after 6 weeks and after 12 weeks compared to baseline, but the scores did not differ between the investigated groups in any of the investigated time points (Figure 3A,B and Figure 4A,B). We also analyzed the difference in the changes in the investigated clinical scores. The total improvement in VISA-P score in the 17° group was 28.2 ± 12.3, with no significant difference compared to the 25° group, which experienced an improvement of 25.7 ± 10.4 (*p* > 0.05). Similar results were found in KOOS (22.8 ± 12.5 vs. 20.6 ± 12.6, *p* > 0.05), in the Lysholm/Tegner Activity Scale (20.1 ± 12.5 vs. 22.8 ± 15.0, *p* > 0.05), and in VAS improvement (63.1 ± 24.0 vs. 55.5 ± 23.8, *p* > 0.05) (Figure 3C,D and Figure 4C,D).

### 3.2. The Improvement in Clinical Scores Was Not Associated with the Demographic Characteristics of the Patients

**Figure 5:**
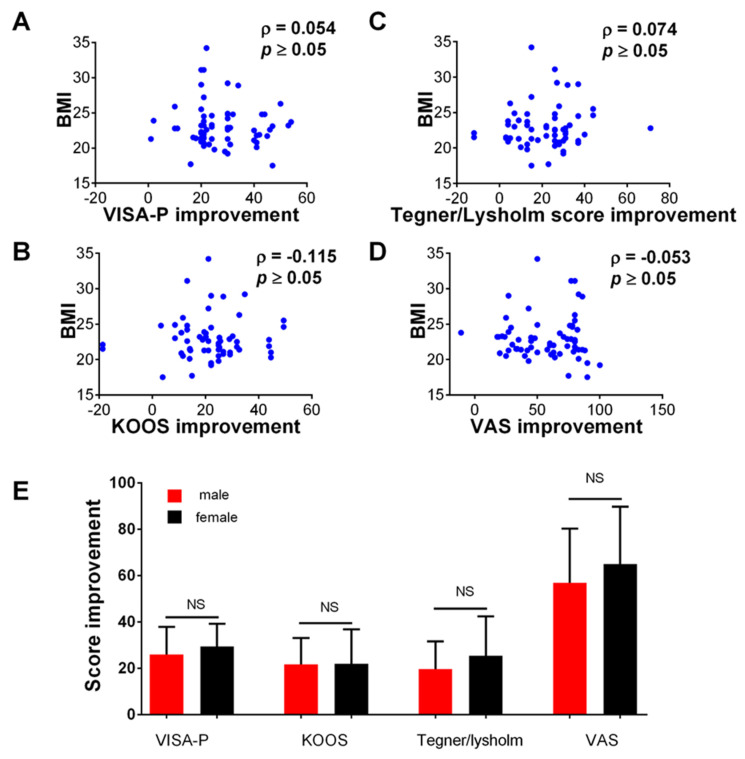
Association between the improvement in the measured clinical scores at the end of the study (12 weeks) and clinical characteristics of the patients. (**A**–**D**) show correlation (Spearman coefficient) between the BMI and VISA-P, KOOS, Lysholm Knee Questionnaire/Tegner Activity Scale, and VAS score, respectively. Dots represent individual values (*n* = 70 patients). In (**E**) comparison between male and female patients in the observed improvement in the clinical score (VISA-P, KOOS, Lysholm Knee Questionnaire/Tegner Activity Scale, and VAS score, respectively) at the end of this study (12 weeks), comparison was made using independent *T*-test. NS—not significant. *n* = 70 patients.

In the final part of our analysis, we were interested in whether improvement in clinical scores can be associated with the demographic characteristics of patients. The analysis showed no significant association between the patients’ BMI and the improvement in VISA-P, KOOS, Lysholm/Tegner Activity Scale, and VAS score (*p* > 0.05 for all analyses, Figure 5A–D). Furthermore, no association was found between the sex of the patients and the clinical improvement (Figure 5E). The improvement in clinical scores was also not associated with the patient’s age or the type of surface of their professional sport (*p* > 0.05 for all analyses,.

## 4. Discussion

In this present research, we compared the effect of two various decline board angles on the clinical outcome of patients treated for patellar tendinopathy by performing eccentric quadriceps exercises. After 6 weeks of exercise, we found a significant improvement in the clinical scores of treated patients, with no significant difference between the patients who performed eccentric quadriceps exercises on the standard 25° decline board and those exercising on the 17° decline board. A smaller additional degree of improvement was visible at the end of the follow-up period (at 12 weeks), but, again, no statistical difference could be detected between the investigated groups, confirming that exercise performed on a 17° decline board provides equal therapeutical benefits to that performed on the standard 25° decline board.

Eccentric quadriceps exercise has been introduced into the therapy of patients with patellar tendinopathy in order to avoid weakening the tendon during the rehabilitation period. The use of a decline board with a decline angle of 25° has been the cornerstone of therapy for the last two decades [30]. The efficacy of the usage of a 25° decline board has been confirmed in various clinically and biomechanically oriented investigations [12,13,31,32]. Overall, the investigations indicate greater benefit of performing squats on the standard 25° decline board than on the flat surface. However, knowledge about the effect of the usage of boards with different angles of decline is still very limited. In biomechanical research, Richards et al. showed the maximum activity of knee extensors when using a 16° decline board, suggesting that the maximum benefit of the exercise can be attained at angles as low as 16° and that no additional mechanical advantage is gained by increasing the angle of declination to 24°. The knowledge that there is a possibility to change the declination angle without a loss in the therapeutic effect would be of significant help to clinicians as patients would be able to choose the angle with which they feel most comfortable, thus ensuring better compliance of the patients during the treatment period. In the present research, we show that the same clinical improvement can be achieved by using a 17° decline board, as evidenced by the improvement of all measured clinical scores: VISA-P score, KOOS score, Lysholm Knee Questionnaire/Tegner Activity Scale, and VAS scale. Neither of the decline angles was superior to the other. In our investigation, patients were randomly assigned to a 17 or 25° group. In future research, it would be interesting to investigate whether better results could be achieved by allowing the patients to personally select the most comfortable decline angle for the exercise.

The significant improvement in the measured clinical scores occurred after 6 weeks of the exercise. The long-term outcomes are of special interest in professional athletes [1,2,32,33]; therefore, we determined the clinical score again after an additional 6 weeks. Importantly, the improvement was still present at that time point, and, in two of the measured scores (VISA-P score and Lysholm Knee Questionnaire/Tegner Activity Scale), we even observed a small but significant degree of further improvement (Figure 1 and Figure 2). We were also interested if the degree of the detected improvement could be predicted by a patient’s age, sex, BMI, or professional sport [34,35]; however, we detected no significant correlation between these variables and improvement in the measured clinical scores.

Our study has several limitations that need to be considered carefully. Although the patients, after enrollment into this study, received clear oral and written instructions to avoid any additional training during the treatment period, it was not possible to control them for their complete compliance with the given instructions during the whole 12-week period. All of the patients declared compliance with the instructions, but there is still a possibility that potential undeclared additional training could have confounded some of the findings for a few patients. As there is a large amount of data showing that exercises performed on the 25° decline board are superior to the exercises performed on the flat surface, it was not possible to include a group of the control patients exercising on the flat surface for ethical reasons. In addition, as this study was primarily oriented to the investigation of the potential clinical benefit, we made no effort to make the biomechanical measurements of the patients to study the activation of particular muscles. The possible influence of other angles of decline was not investigated, and it remains to be investigated in future investigations.

We have shown that the usage of a 17° decline board does not affect the beneficial therapeutic effects of the eccentric exercises, but there are still many unanswered questions that should be studied in future investigations. In our study, we randomized the patients into the 17° decline board and the 25° decline board groups, but further biomechanical studies are necessary to try to identify predictors of ideal muscular activation such as height, weight, or type of the sport. These findings might lead to the personalization of the treatment by enabling physicians to choose the best angle of decline for the particular patient. A bioinformatic approach based on deep learning to analyze collected data might provide additional help to achieve these goals [36].

## 5. Conclusions

For the treatment of patients with patellar tendinopathy, we have shown that the performance of exercises on the 17° decline board is an equally good treatment option as the performance of exercises on the standard 25° decline board. Both treatment options provide similar short-term and midterm benefits in the improvement of pain and clinical scores. The improvement in the clinical scores does not depend on age, sex, BMI, or the professional sport of the patient. Our findings encourage a change in the decline angle of the board in the case of a patient’s discomfort in order to achieve better compliance without affecting the recovery.

## Figures and Tables

**Table 1:** Demographic characteristics of the patients and clinical scores at the time of the inclusion into this study.

	17° Decline Board	25° Decline Board	*p* Value
Age (years)	25.03 ± 6.8	24.11 ± 6.97	0.58
Sex			
Male	26 (74.3%)	23 (65.7%)	0.603
Female	9 (25.7%)	12 (34.3%)	
Body weight (kg)	74.17 ± 13.73	70.94 ± 11.25	0.286
Height (cm)	177.71 ± 6.31	176.97 ± 6.89	0.639
BMI	23.47 ± 3.19	22.65 ± 2.78	0.255
VAS score	76.66 ± 10.26	76.49 ± 9.01	0.941
VISAP score	53.54 ± 12.29	52.77 ± 12.33	0.794
Lysholm/Tegner	67.46 ± 12.92	60.8 ± 17.58	0.076
KOOS score	64.73 ± 11.67	62.42 ± 14.44	0.405
Surface			
Grass	22 (62.9%)	19 (54.3%)	0.257
Wood	10 (28.6%)	8 (22.9%)	
other	3 (8.6%)	8 (22.9%)	

## Data Availability

Data are contained within this article. Any additional data are available from the corresponding author upon reasonable request.
